# Shrinking the Lymphatic Filariasis Map of Ethiopia: Reassessing the Population at Risk through Nationwide Mapping

**DOI:** 10.1371/journal.pntd.0004172

**Published:** 2015-11-05

**Authors:** Maria P. Rebollo, Heven Sime, Ashenafi Assefa, Jorge Cano, Kebede Deribe, Alba Gonzalez-Escalada, Oumer Shafi, Gail Davey, Simon J. Brooker, Amha Kebede, Moses J. Bockarie

**Affiliations:** 1 Centre for Neglected Tropical Diseases, Department of Parasitology, Liverpool School of Tropical Medicine, Pembroke Place, Liverpool, United Kingdom; 2 Ethiopian Public Health Institute, Addis Ababa, Ethiopia; 3 London School of Hygiene & Tropical Medicine, London, United Kingdom; 4 Brighton and Sussex Medical School, Falmer, Brighton, United Kingdom; 5 School of Public Health, Addis Ababa University, Addis Ababa, Ethiopia; 6 Faculty of Infectious and Tropical Diseases, Rey Juan Carlos University, Madrid, Spain; 7 Federal Ministry of Health, Addis Ababa, Ethiopia; George Washington University, UNITED STATES

## Abstract

**Background:**

Mapping of lymphatic filariasis (LF) is essential for the delineation of endemic implementation units and determining the population at risk that will be targeted for mass drug administration (MDA). Prior to the current study, only 116 of the 832 woredas (districts) in Ethiopia had been mapped for LF. The aim of this study was to perform a nationwide mapping exercise to determine the number of people that should be targeted for MDA in 2016 when national coverage was anticipated.

**Methodology/Principal Finding:**

A two-stage cluster purposive sampling was used to conduct a community-based cross-sectional survey for an integrated mapping of LF and podoconiosis, in seven regional states and two city administrations. Two communities in each woreda were purposely selected using the World Health Organization (WHO) mapping strategy for LF based on sampling 100 individuals per community and two purposely selected communities per woreda. Overall, 130 166 people were examined in 1315 communities in 658 woredas. In total, 140 people were found to be positive for circulating LF antigen by immunochromatographic card test (ICT) in 89 communities. Based on WHO guidelines, 75 of the 658 woredas surveyed in the nine regions were found to be endemic for LF with a 2016 projected population of 9 267 410 residing in areas of active disease transmission. Combining these results with other data it is estimated that 11 580 010 people in 112 woredas will be exposed to infection in 2016.

**Conclusions:**

We have conducted nationwide mapping of LF in Ethiopia and demonstrated that the number of people living in LF endemic areas is 60% lower than current estimates. We also showed that integrated mapping of multiple NTDs is feasible and cost effective and if properly planned, can be quickly achieved at national scale.

## Author Summary

About 1.4 billion people are believed to be living in areas where Lymphatic filariasis (LF) is actively transmitted. However, the distribution of this disfiguring mosquito-borne parasitic disease and the true population at risk that can be targeted for treatment have not been defined for all endemic countries. By 2013, Ethiopia had not delineated the majority of the endemic implementation units that can be targeted for MDA. Here, we present the results of a nationwide mapping exercise conducted in 2013 to determine the number of people that should be targeted for treatment in 2016 when nationwide treatment coverage is expected. We adopted a two-stage cluster purposive sampling method for the integrated mapping of LF and podoconiosis in seven regional states and two city administrations. Using a WHO mapping strategy for LF, based on sampling 100 individuals per community ICT positive individuals (ICT+) and two purposely selected communities per district, we examined 130 166 people in 1315 communities in 658 districts. Only 140 people were found to be positive for LF antigen in 89 different communities. According to WHO guidelines, 75 of the 658 districts surveyed in the 9 regions were found to be LF endemic. Including the 37 endemic *Woredas* identified enprior to this study, 112 *woredas* across the country are known to be endemic for the disease with 11 580 010 people exposed to infection. However 6 190 482 of those resided in *woredas* where our survey results were borderline with only one ICT positive individual identified. We have demonstrated that the number of people living in areas of active LF transmission is at least 60% lower than current WHO estimates of 30 million. We also showed that integrated mapping of multiple NTDs is feasible and cost effective. However, the sensitivity of the diagnostic test used for LF is less than 100% and the identification of a single ICT positive adult may not provide evidence of disease transmission. Based on these limitations, and in addition to the restricted geographical representation of just two sites within a *woreda*, we recommend conducting research in the 45 *woredas* with borderline results (one ICT+) to shrink the denominator even further.

## Introduction

Lymphatic filariasis (LF) is a mosquito-borne neglected tropical disease (NTD) associated with debilitating conditions that affect at least 40 million people worldwide [1]. Chronic LF is manifested in the form of acute dermatolymphangioadenitis, lymphoedema, elephantiasis of the limbs and hydrocele. These disfiguring conditions are stigmatizing and affect mobility leading to impairment in educational and employment opportunities. In Africa, the causative agent of LF, *Wuchereria bancrofti*, is transmitted by many species of mosquitoes belonging to the *Anopheles*, *Culex* and *Mansonia* genera. *Anopheles* species are the main vectors LF in Ethiopia [2]. LF was recognised in 1993 as an eradicable disease [3, 4] and systematic efforts aimed at eliminating the disease were initiated in 1997 through a World Health Assembly Resolution (WHA 50.29) calling for the elimination of the disease as a public health problem in all endemic countries. To accelerate the elimination process, pharmaceutical companies, donors, governments and other partners renewed their commitments in 2012 through London Declaration, to control, eliminate or eradicate ten NTDs including lymphatic filariasis by 2020 [5]. The renewed commitment was to complete mapping, ensure the continued supply of drugs for preventive chemotherapy, advance research and scale up the implementation of control interventions. Only communities that have been shown to be endemic through mapping are targeted for treatment.

In March 2011, the endemicity status of 9 countries historically considered to be endemic but with no current evidence for active transmission (Burundi, Cape Verde, Costa Rica, Mauritius, Rwanda, Seychelles, Solomon Islands, Suriname, and Trinidad and Tobago) was reviewed. The outcome of the review led WHO Strategic and Technical Advisory Group on Neglected Tropical Diseases to reclassify all 9 of them as non-endemic [6].

WHO currently estimates that 1.4 billion people live in areas where LF is actively transmitted, nevertheless in 2013, 13 of the 73 countries where LF is known to exist had not delineated all or the majority of the endemic implementation units (IU) for mass drug administration (MDA) to a defined population at risk, including Ethiopia [1]. Moreover as more sensitive diagnostic and transmission monitoring tools become available and mapping progresses, the country specific estimates for the number of people living in areas with active transmission will be reassessed to determine the amount of medicines required for community-wide treatment [7].

According to WHO estimates, in Africa, about half (48.5%) of the 464 million people exposed to LF reside in the four high burden countries of Democratic Republic of Congo (DRC) (49 million), Ethiopia (30 million), Nigeria (109 million) and Tanzania (45 million) [8]. With the exception of Tanzania, the high burden countries in Africa are yet to complete mapping of all implementation units. In Ethiopia only 116 of the 832 districts in the country have so far been mapped for the disease [9].

The four sequential steps, recommended by WHO for the implementation of MDA-based interventions, begin with mapping [10]. Mapping is the systematic epidemiological assessment of all geographical areas to determine if a disease is actively transmitted in each area. The implementation unit (IU), typically the district or second administrative division of government, is the basic survey unit that will inform if MDA is required. In Ethiopia, the IU corresponds to the *woreda* (district).

Historically, the known distribution of LF in Ethiopia was restricted to the lowlands of the south-western regions, especially in Gambella Region [11–14]. In 2008, the Carter Center and Addis Ababa University conducted surveys in 112 districts in western Ethiopia where onchocerciasis is also endemic [15]. The main objective of the 2008 mapping surveys was to determine the presence or absence of LF infection in districts that were under ivermectin treatment in order to add albendazole to the preventive chemotherapy package on those that were endemic for LF. As recommended by WHO, the selection of villages was biased towards finding LF infection. The WHO Operational Guidelines for Mapping of Bancroftian Filariasis in Africa informed the sampling procedures and the *woreda* (district) was defined as the unit for determining LF endemicity. About 100 people were tested in each selected *woreda*, and *woredas* where one or more positives were found were classified as endemic. Thirty-four of the 112 districts, with a population of 1 547 685 in 2007, were found to be endemic and MDA against LF commenced in Ethiopia in 2009 [16].

To date, less than 2 million people have been targeted for treatment representing 5.3% national coverage with an estimated 30 million people at risk as the denominator. In this paper we present the results of the nationwide mapping exercise carried out in 2013 to inform the number of people that should be targeted for MDA in 2016 when national treatment coverage is expected. We also discuss the significance of the reduction of the denominator, and the impact of shrinking the LF map in Ethiopia and other high-burden countries to accelerate the achievement of the 2020 goal of LF elimination.

## Materials and Methods

### Study area

With 30 million people estimated to be living in areas where LF is transmitted, Ethiopia ranks fourth in Africa with regard to number of people at risk of infection [8]. Located in the horn of Africa, Ethiopia is a landlocked country with a surface area of some 1.1 million square kilometres. The estimated population for 2014 is 86.6 million with over 45% of its people aged 15 years or older [17]. Population estimates used in this study are based on the projections by the Central Statistical Agency of the government of Ethiopia published in 2013, to the *woreda*, level for the years 2014, 2015, 2016 and 2017 (http://www.csa.gov.et/images/general/news/pop_pro_wer_2014-2017_final).

Ethiopia is characterised by great geographical diversity; ranging from the deserts along the eastern border to the tropical forests in the south to extensive Afromontane in the northern and south-western regions. The topographic features range from the highest peak at 4 550 metres above sea level to 110 metres below sea level. This wide diversity of terrain has given rise to a huge variation in climate, soils and natural vegetation that produces unique biological niches suitable for different flora and fauna.

The federal administrative divisions in the country are presented as 9 regions and 2 city administration councils. The regions are sub-divided into zones and *woredas* but city administrations are only divided into *woredas*. In total, there are currently 832 *woredas* in Ethiopia which are further divided into well-defined communities called *kebeles* [17]. Prior to the current study, the endemicity and distribution of LF in Ethiopia had only been established for 116 districts in parts of the regions of Gambella, Benishangul-Gumuz, Southern Nations Nationalities and Peoples (SNNPR), Amhara and Oromia[15]. Of the remaining 716 districts, only the 710 *woredas* outside the national capital, Addis Ababa, were targeted for mapping during this survey. Addis Ababa was excluded from this survey because of the difficulties associated with mapping urban areas using purposive sampling (rural to urban migration and population movements inside the capital make it difficult to select areas of higher risk). Further investigation will be required using more appropriate methods to assess transmission in non rural areas like the national capital (for example xenomonitoring and school cluster random sampling used during transmission assessment surveys).

### Study design and sampling

The details of the survey design, procedures and experiences of conducting the survey are provided elsewhere [18]. In brief, a two-stage cluster purposive sampling was used to conduct a community-based cross-sectional study of mapping two diseases, LF and podoconiosis, in 7 regional states (Tigray, Affar, Amhara, Oromiya, Somali, Southern Nations Nationalities and Peoples (SNNP), and Harari) and 2 city administrations (Addis Ababa and Dire Dawa Administration Councils). Surveys were not conducted in the Benishangul-Gumuz and Gambella regions because they had previously been mapped for LF in 2008. Surveys for podoconiosis and LF were conducted simultaneously using a coordinated mapping strategy between June and October 2013. Initially, *kebeles* (communities) considered to be at high risk for LF were identified based on health facility records of morbidity associated with LF, presence of diseased individuals and suitability for vector mosquito breeding. Two *kebeles* in each *woreda* were purposely selected using the WHO mapping strategy for LF based on sampling 100 individuals per community and two purposely selected communities per IU (i.e. *woreda* in Ethiopia) [10]. The primary sampling unit for the LF survey was the *kebele* and consenting individuals were selected systematically with a random start from those 15 years or older. Social mobilization was conducted one day prior to the survey using health extension workers. During the awareness campaign, an attempt was made to inform every adult in the community through a house to house visit that a survey was to be conducted, and that they were invited to participate. We have previously described in detail the survey protocols and sampling procedures used in the study including the lessons learnt from the integrated mapping of LF and podoconiosis in Ethiopia [18].

### LF diagnosis

The presence of LF infection was determined by the immunochromatographic card test (ICT) markedted as Binax NOW Filariasis card test (Alere Inc., Scarborough, ME) which detects circulating filarial antigen (CFA) as described in the WHO guidelines [10] and in a recent review [7]. Fingerprick blood from individuals were transferred to an ICT card using a calibrated capillary tube and results were read 10 minutes after closing the card, following manufacturer’s instructions. Recombinant *W*. *bancrofti* antigen was used as a positive control to confirm the quality of the ICT cards. The field team spent several days outside the main towns and ICT kits were refrigerated while in storage in central points in hospitals and regional laboratories across the country. ICT test results were recorded on the ICT card and entered in a database on a smartphone platform (see below).

### Questionnaires and clinical examinations

The survey forms used included questions regarding general demographics and LF control activities. In addition to collecting information on study site, region, zone, *woreda* (district) and *kebele* (community), questions were asked about sleeping under a bednet the previous night, deworming and treatment with ivermectin and or albendazole in the past year. Participants were not clinical examined for hydrocele but were asked to self-report if they had hydrocele. Signs of lymphedema in the lower extremities were recorded by trained nurses during physical examination in the interviews. For individuals with lymphedema, an algorithm was used to differentiate between LF and podoconiosis as described elsewhere [18].

### Data entry and analysis

Motorola Atrix HD smartphones with GPS capabilities, long life batteries and an android application were used for data collection for the coordinated mapping of LF and podoconiosis, as detailed elsewhere [18]. Data were then downloaded in Excel format and imported to STATA 13.0 (Stata Corporation, College Station, TX) for further cleaning and analysis. Maps of infection distribution were generated using ArcGIS 10.2 (ESRI, California). Hierarchal data were collected using separate surveys for community level information and for individual level information. The different survey data sets were later linked to produce a complete analytic database. The community survey forms included population counts and information about community-wide treatment of LF and other deworming activities in the past year. Detailed description and results of the demographic and podoconiosis surveys have been published separately [18].

An endemic district for lymphatic filariasis was defined using a threshold for LF infection of at least one infected individual in either of the two selected communities in each *woreda*. This definition is consistent with the WHO definition of endemic IUs (being those where any subunit of the district has an antigenemia or microfilaremia rate of 1% or greater). The relationship between infection and risk factors was investigated using univariate logistic regression, adjusted for clustering at the community level. As one of the objectives of this study is to determine the population at risk in 2016, when national MDA coverage is anticipated, we used the populations projections provided by the Federal Democratic Republic of Ethiopia Central Statistical Agency (http://www.csa.gov.et/images/general/news/pop_pro_wer_2014-2017_final). Using the government projection figures at the Woreda level encouraged communication between the WHO country office, the Federal MOH, Regional Health Bureaus and the NTD programmes in preparing requests for medicines for MDA.

### Ethical approval and consent procedures

Ethical clearance for the study was granted by the Research Ethics Committee of the Liverpool School of Tropical Medicine (Research Protocol 12.22), the Institutional Review Board of the Medical Faculty, Addis Ababa University and the ethics committee at the Ethiopian Public Health Institute (EPHI). Details on the consent procedures are provided elsewhere [18]. Briefly, individual written informed consent was obtained from each participant aged 18 years and above. Consent for younger survey participants, 15 to 18 years of age was obtained from their parents/guardian and the participant themselves provided informed assent. The response rate was 98.6%. For those with lymphoedema, health education was given about how to manage their condition and prevent any disability.

## Results

Overall, 130 116 people were examined in 1315 communities in 658 *woredas* (Table 1). Fifty-two *woredas*, mostly in the Somali region, could not be surveyed because they were not easily accessible due to major logistical challenges encountered by the survey teams. The median age of individuals was 34 years (Inter-quartile range (IQR): 25–46), but this ranged from 30 (IQR: 23–40) years in Afar Region to 39 (IQR: 27–50) years in Somali Region, with age range between 15 and 100 years. Many survey participants had been resident in their respective regions for more than 30 years (median: 30, IQR:22–45) but very few had attained education levels beyond the primary school grade. The mean number of individuals surveyed per community was 99 (sd: 9.3).

**Table 1:** The number of implementation units (IUs) per region and populations at risk for LF determined by immunochromatographic card tests (ICT) in 11 regions/zones in Ethiopia. The 75 endemic IUs identified during the 2013 mapping surveys included 45 with borderline ICT results based on one ICT+ only. The updated number of endemic IUs, including results from previous surveys, and the projected population at risk in 2016 are presented in the last two columns. Populations for 2016 are based on population projection estimates provided by the Federal Democratic Republic of Ethiopia Central Statistical Agency: http://www.csa.gov.et/images/general/news/pop_pro_wer_2014-2017_final.

**Region**	No. of IUs	Population of IUs	No. of IUs mapped	No. of people examined for LF	No. positive for LF	No. of endemic IUs (1 ICT+)	Population of endemic IUs (1 ICT+)	No. of endemic IUs (>1 ICT+)	Population of endemic IUs (> 1 ICT+)	No. of endemic IUs (all surveys)	Total Population at risk for LF
**Tigray**	47	5151998	46	9 164	8	4	469503	1	111993	5	581496
**Afar**	33	1769002	32	6 289	1	1	41360	0	0	1	41360
**Amhara**	153	20769985	140	27 721	19	13	2458242	3	240171	19	2973020
**Oromia**	308	34575008	243	48 003	62	19	2166826	13	1353217	36	3990525
**Somali**	72	5598002	49	9 583	0	0	0	0	0	0	0
**Benshangul G**	21	1033999	0	0	0	0	0	0	13	13	604592
**SNNPR**	158	18719008	128	25 354	49	7	1036930	13	1371534	30	3176186
**Gambella**	13	422002	0	0	0	0	0	0	0	7	194499
**Harari**	9	240000	9	1 800	1	1	17621	0	0	1	18332
**Dire Dawa**	8	453000	7	1 402	0	0	0	0	0	0	0
**Addis Ababa**	10	3352000	4	800	0	0	0	0	0	0	0
**Total**	**832**	**92084004**	**658**	**130 166**	**140**	**45**	**6 190 482**	**30**	**3 076 928**	**112**	**11 580 010**

### LF endemicity and distribution

**Fig 1:**
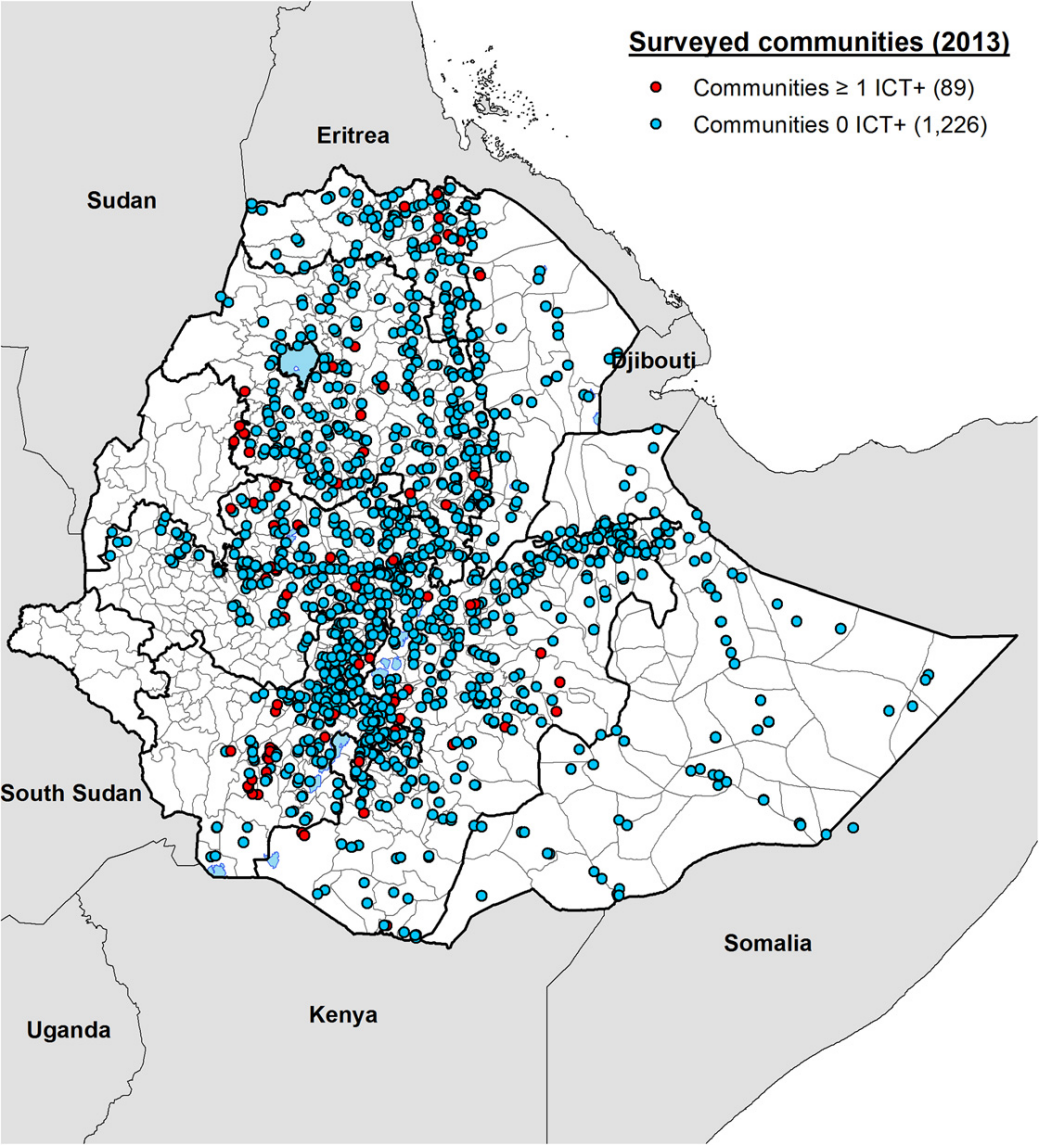
Map showing the locations of 1315 communities surveyed in Ethiopia during the mapping project in 2013. Eighty-nine communities in which one or more persons out of 100 individuals tested were found to be positive for circulating filarial antigen (CFA) are shown in red. Communities where no positive individuals were identified after testing approximately 100 adults are marked in blue.

In total, 140 people were found to be positive for CFA by ICT performed in 89 different communities (Table 1). The median age of CFA-positive individuals was 35.8 (IQR: 25–41.5) years with range between 15 and 80 years. Fig 1 shows the location of all 1315 communities that were surveyed and the distribution of the 89 communities with CFA positive individuals. Communities with CFA positive individuals were observed in all the regions surveyed with the exception of Somali and the two city administration councils of Addis Ababa and Dire Dawa. Only one community was shown to be positive in the Afar Region. Communities with positive individuals were equally distributed in the northern and southern parts of the country. The CFA positivity rates for males and females in the endemic *woredas* were 0.8% (60/7443) and 1.1% (80/7429), respectively.

**Fig 2:**
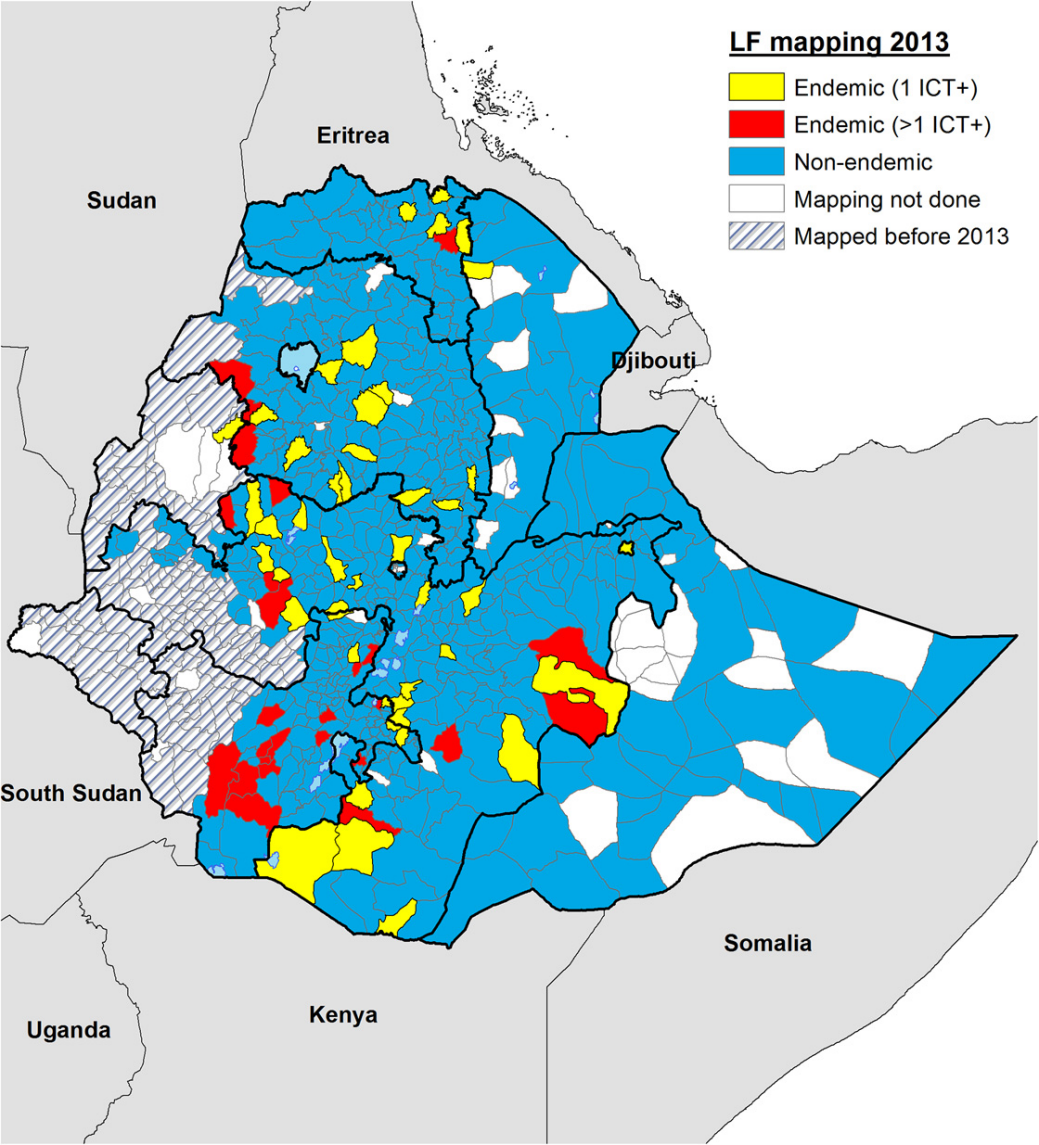
Map showing the distribution of endemic and non-endemic implementation units (UIs) or *woredas* identified during the 2013 mapping surveys in Ethiopia. After completing surveys in 658 implementation units, 75 were classified as endemic based on finding at least one infected person after testing approximately 200 individuals in two different communities. Forty-five of the endemic IU, shown in yellow had borderline endemicity with only one CFA positive person identified (1 ICT+). The 30 IUs with two or more CFA positive individuals (>1 ICT+) are shown in red.

Based on WHO guidelines, whereby *woredas* with any CFA positive individual are classified as endemic [10], 75 of the 658 *woredas* surveyed in the nine regions were found to be endemic (Fig 2), including 45 *woredas* where only one ICT positive was observed (in yellow). The estimated population for these 75 endemic *woredas* is 9 267 410, based on the projected population for 2016 as indicated previously.

**Fig 3:**
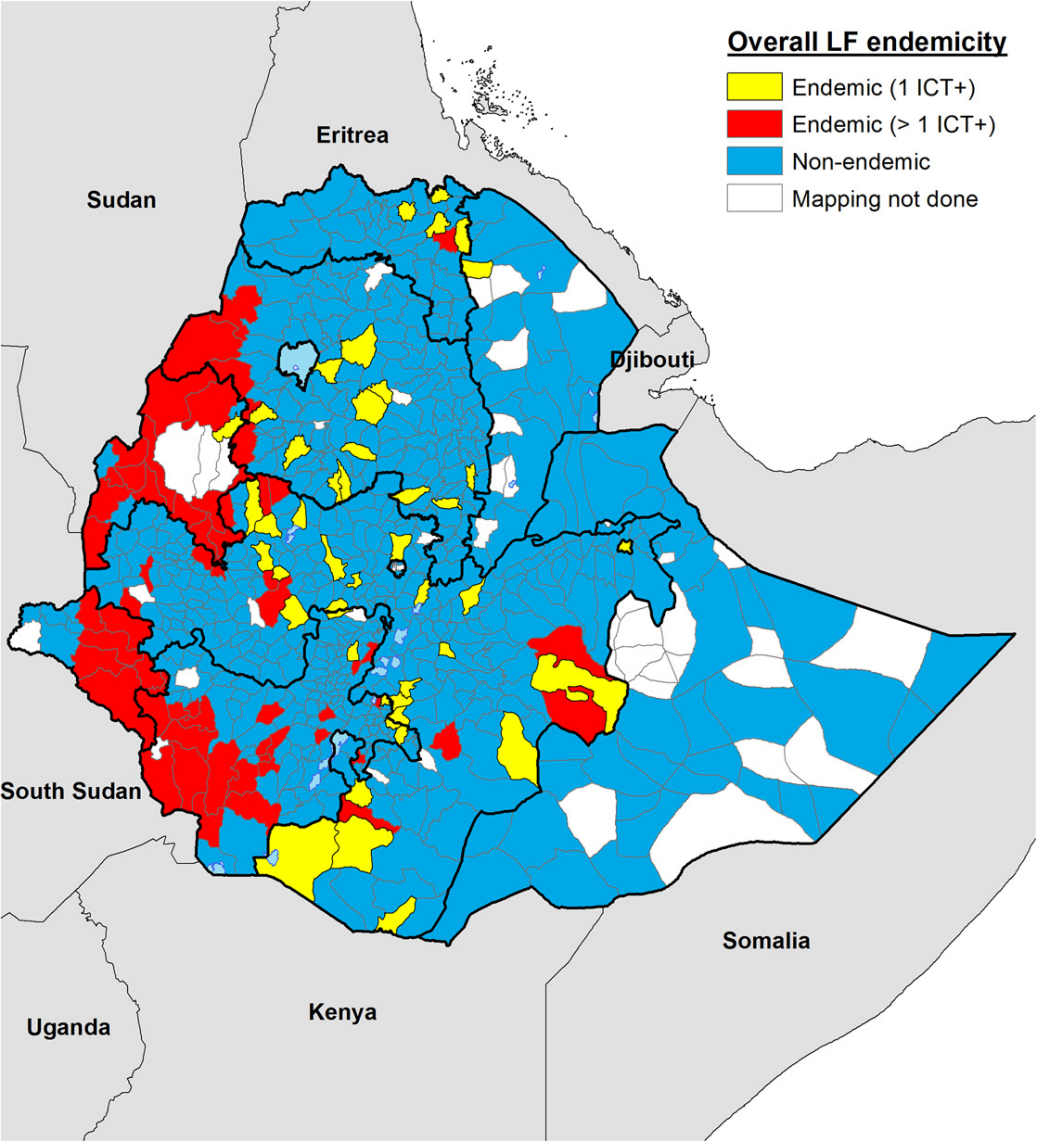
Current LF endemicity map for Ethiopia for all IUs surveyed to date. Endemic IUs are shown in red (>1 ICT+) and yellow (1 ICT+) depending on the ICT results. Non-endemic IUs are shown in blue leaving only 58 districts yet to be mapped.

Fig 3 shows the updated endemicity map for Ethiopia incorporating data from previous published mapping surveys [15]. The estimated total population at risk of LF for the 112 endemic districts in Ethiopia is 11 580 010, including 6 190 482 from the 45 *woredas* where only 1 ICT positive individual was identified during the survey, being therefore on the margin of the endemicity threshold. This number includes the 75 newly-discovered endemic *woredas* and the 37 previously known to be endemic.

### Relationship between reported interventions and CFA positivity

Individuals who were residing in the endemic *woredas* were interviewed about interventions that might impact exposure to LF and the likelihood of becoming CFA positive. In the 75 newly-identified endemic *woredas*, out of 14 676 people who responded to questions about previous treatment with an LF medication, only 499 (3.4%) reported that they had received treatment for LF. We found no significant association between CFA positivity rates and reported previous treatment for LF. Similarly, there was no association between reported deworming and a CFA negative outcome (OR 1.14, 95% confidence interval (CI) 0.77–1.69). We also failed to demonstrate an association between bednet usage and being negative for CFA (OR 1.04, 95% CI 0.74–1.45).

### Relationship between LF morbidity and CFA positivity

The risk of presenting with lymphedema at any stage was almost double (OR 1.80, 95% CI 1.68–1.93) for those living in an endemic *woreda*. Hydrocele was self-reported by individuals and not verified by health personnel which represents a potential limitation in accuracy of any association found. Nevertheless, we found almost twice the risk (OR: 1.91, 95%CI: 1.4–2.5) of reporting hydrocele among males living in endemic compared to non-endemic areas.

## Discussion

Our surveys increased the number of *woredas* mapped for LF in Ethiopia from 116 to 774 representing 93% of the mapping required. With only 58 *woredas* out of 832 remaining to be mapped, the nationwide mapping of LF in Ethiopia indicates that 112 districts across the country are endemic for the disease. The projected population that will require treatment in these endemic districts in 2016, when national MDA coverage is anticipated, is 11 580 010. The population at risk for LF, determined through our surveys, is 60% lower than the current WHO estimates [8]. Our findings are important in providing well-informed nationwide estimates of the distribution of LF and demonstrating the value of coordinated mapping of multiple diseases [18] that can result in significant cost savings for national NTD programmes that currently rely on disease-specific mapping protocols [19].

Historically, LF has been reported as having limited distribution in the southwest of the country. The disease was first detected in a native Ethiopian in 1937 [13] but it was never reported as widely distributed outside the Gambella area, where examination of 1 ml of diurnal blood in 82 adults in 1971 revealed a 24% microfilaraemia rate[13]. A more recent survey in Gambella (1993) confirmed the high transmission intensity of *W*. *bancrofti* in this area, with an average microfilaraemia rate of 20.7% in the population surveyed at two communities adjacent to the Baro river [20]. Hydrocele and elephantiasis were however uncommon in the Gambella area and studies carried out in 1976 [21] ruled out the involvement of *W*. *bancrofti* in the occurrence of elephantiasis in the highlands, where this condition had previously been reported [22, 23]. Entomological investigations in the Gambella area showed that *Anopheles gambiae s*.*l* (probably *An*. *arabiensis*) and *An*. *funestus* were the main vectors, with no evidence for the involvement of the culicine mosquitoes despite the high biting rates [13]. A total of 3 228 *Mansonia* mosquitoes, vectors of LF in West Africa [24], were dissected but none was found to be infected. However, the *W*. *bancrofti* infectivity rates in *An*. *gambiae s*.*l* (0.24%) and *An*. *funestus* (0.35%) were lower than what was reported in other parts of East Africa at the time, including Kenya [25], and Tanzania [26], suggesting that the intensity of LF transmission in the Gambella area was relatively low despite the high MF rate shown in adults. The low infectivity rates for the vectors in Ethiopia were probably related to the low intensity of infections observed because 9 of the 20 infected persons were low density MF carriers with less than 6 mf/ml of blood. Only 3 of the 20 infected persons harboured more than 100 mf/ml of blood [13]. In his review, Southgate [27] concluded that *Anopheles* mosquitoes are poor vectors when their blood-meal source is presented by low density MF carriers.

Cano and co-workers [28] have recently modelled the spatial limits of LF transmission for Africa using *boosted regression trees* (BRT) approach over a suite of environmental and climatic data and available mapping data, including data generated by this study,. The environmental suitability for LF was shown to be very low for over 80% of Ethiopia and moderate for narrow bands in the western and south western parts of the country [28, 29]. Recent predictive maps of LF prevalence in Africa, performed using Bayesian geostatistics modelling approach, presents Ethiopia as one of the lowest burden countries in Africa with an estimated infection rate of 2.8% for 2000 and disease distribution limited to less than 20% of the country [28–30].

It is not clear how the WHO estimate of 30 million people at risk for LF in Ethiopia was derived. Based on the high proportion of low density MF carriers in endemic areas, consistent prediction of transmission probability in less than 20% of the landmass by predictive models and the relative inefficiency of the LF vectors in Ethiopia [28–30], it is highly unlikely that 30 million people are at risk for LF in Ethiopia. The wide distribution of malaria, which is transmitted by LF vectors, and lymphedema associated with podoconiosis may have historically suggested a wider distribution of the disease than demonstrated by the current study. Additional entomological studies to improve understanding of mosquito distribution and LF transmission dynamics would be beneficial to inform public health policies.

There are three important limitations of the mapping methods used on this research. Firstly, mapping LF has the objective of identifying areas where transmission is active and therefore preventive chemotherapy is required. In 45 out of 658 IUs surveyed during this exercise, only one positive individual for CFA was identified. Fig 2 shows in yellow the location of these 45 districts with ‘borderline’ results. ICT cards are not 100% sensitive and specific, furthermore antigenemia may remain positive even after infection has been cleared and individuals can move from one district to another, therefore it is arguable whether one positive individual by ICT equates to active transmission in an IU. Operational research in these 45 borderline results districts is therefore required to assess the use of more robust tools to determine whether transmission of LF is truly active and MDA necessary. Greater geographical representation will be required in the districts in which purposive sampling has failed to identify a significant number of positive individuals. Demonstrating that MDA is not needed in any of these 45 IUs would potentially represent important cost savings in terms of cost of drugs, distribution, supervision, coverage surveys, impact assessments and surveillance post MDA. Secondly, although we found twice the risk of presenting with clinical symptoms for those living in endemic *woredas*, due to the similarities between LF and podoconiosis morbidity, selection of sites for mapping may have failed to identify areas where LF transmission is active. Thirdly, we cannot completely rule out ongoing transmission in other communities within a *woreda* just because no CFA-positive individuals have been found in the two communities selected for the mapping survey. The selection of these communities relies on the evidence or suspicion of ongoing transmission, normally based on the report of clinical cases or environmental suitability for transmission. In low endemicity settings, few clinical cases are expected and therefore hot spots of LF transmission can easily be overlooked by the health public system. According to our results, confirmation using more statistically robust methods and a larger sample size with greater geographical representation will be required in those districts declared non-endemic to certify that they are completely free of LF before WHO can provide certification that Ethiopia has eliminated LF. Moreover, the sensitivity of the diagnostic test used for LF in this study is less than 100% and the identification of a single ICT positive adult may not provide evidence of disease transmission. Based on these limitations, in addition to the restricted geographical representation of just two sites within a *woreda*, we recommended conducting operational research in the 45 *woredas* with borderline results (one ICT+) to shrink the denominator even further. This recommendation was endorsed by the WHO AFRO Regional Programme Review Group (RPRG) in 2014 and submitted to the Federal Ministry of Health through the WHO country office for action.

The present study was conducted as part of an integrated mapping project for LF and podoconiosis [18] and the nationwide survey was completed within three months. During that period we visited 1315 communities and examined 130 166 individuals in 658 *woredas* across Ethiopia. A detailed description of the integrated mapping of lymphatic filariasis and podoconiosis and the lessons learnt from the exercise have been published separately [18]. Mapping the overlap of multiple NTDs in an implementation unit is critical for informing strategies for interventions, morbidity management and disability prevention. This is particularly important in areas in which LF may be co-endemic with loiasis, malaria, onchocerciasis or podoconiosis. The Ethiopian government planning budgets for disease-specific mapping of LF and podoconiosis in 710 planned districts, including diagnostics, training, field work, data management, supervision, were $1 212 209 and $1 211 664 respectively, but the actual financial cost of our coordinated mapping of LF and podoconiosis was only $1 291 400 for 658 districts [18]. This significant reduction in total cost for mapping both diseases was achieved through savings in the areas of team training, supply chain management and travel. The total cost of uncoordinated disease specific mapping for the two diseases was 1.9 times as high as the integrated survey approach [18].

In conclusion, with 93% of the *woredas* in Ethiopia mapped, the LF map of Ethiopia is almost complete. In the process, we demonstrated that the number of people living in LF endemic areas is 60% lower than previous estimates. We also showed that integrated mapping of multiple NTDs is feasible and cost-effective and if properly planned, can be quickly achieved at national scale. This is very encouraging for accelerating the mapping of NTDs in Africa, estimating the true population at risk of LF, scaling up to reach 100% geographical coverage of MDA and banishing lymphatic filariasis to history.

## Supporting Information

S1 Checklist STROBE Checklist. (DOC)
